# DNA Salivary Methylation Levels of the ACE2 Promoter Are Not Related to ACE2 (*rs2285666* and *rs2074192*), TMPRSS2 (*rs12329760* and *rs2070788*) and ACE1 *rs1799752* Polymorphisms in COVID-19 Survivors with Post-COVID-19 Condition

**DOI:** 10.3390/ijms26052100

**Published:** 2025-02-27

**Authors:** César Fernández-de-las-Peñas, Gema Díaz-Gil, Antonio Gil-Crujera, Stella M. Gómez-Sánchez, Silvia Ambite-Quesada, Juan Torres-Macho, Pablo Ryan-Murua, Ana I. Franco-Moreno, Oscar J. Pellicer-Valero, Lars Arendt-Nielsen, Rocco Giordano

**Affiliations:** 1 Department of Physical Therapy, Occupational Therapy, Rehabilitation and Physical Medicine, Universidad Rey Juan Carlos, 28922 Alcorcón, Spain; silvia.ambite.quesada@urjc.es; 2 Center for Neuroplasticity and Pain (CNAP), Sensory-Motor Interaction (SMI) Center, Department of Health Science and Technology, Faculty of Medicine, Aalborg University, DK-9220 Aalborg, Denmark; lan@hst.aau.dk (L.A.-N.); rg@hst.aau.dk (R.G.); 3 Research Group GAMDES, Department of Basic Health Sciences, Universidad Rey Juan Carlos, 28933 Madrid, Spain; gema.diaz@urjc.es (G.D.-G.); antonio.gil@urjc.es (A.G.-C.); stella.gomez@urjc.es (S.M.G.-S.); 4 Department of Internal Medicine, Hospital Universitario Infanta Leonor-Virgen de la Torre, 28031 Madrid, Spain; juan.torresm@salud.madrid.org (J.T.-M.); pabloryan@gmail.com (P.R.-M.); anaisabel.franco@salud.madrid.org (A.I.F.-M.); 5 Department of Medicine, School of Medicine, Universidad Complutense de Madrid, 28040 Madrid, Spain; 6 CIBER de Enfermedades Infecciosas (CIBERINFEC), ISCIIII, 28040 Madrid, Spain; 7 Image Processing Laboratory (IPL), Universitat de València, Parc Científic, 46100 Paterna, Spain; oscar.pellicer@uv.es; 8 Department of Gastroenterology & Hepatology, Mech-Sense, Clinical Institute, Aalborg University Hospital, DK-9000 Aalborg, Denmark; 9 Steno Diabetes Center North Denmark, Clinical Institute, Aalborg University Hospital, DK-9000 Aalborg, Denmark; 10 Department of Oral and Maxillofacial Surgery, Aalborg University Hospital, DK-9000 Aalborg, Denmark

**Keywords:** methylation, polymorphism, ACE2, TMPRSS2, ACE1, long COVID

## Abstract

Genetics and epigenetics are mechanisms proposed for explaining post-COVID-19 condition. This secondary analysis aimed to investigate if DNA methylation levels of the ACE2 promoter are different depending on the genotype of five COVID-19-related polymorphisms in individuals who had been previously hospitalized due to SARS-CoV-2 infection. We collected non-stimulated saliva samples from 279 (48.7% female, age: 56.0 ± 12.5 years) previously hospitalized COVID-19 survivors. The participants self-reported for the presence of post-COVID symptomatology that started after the infection and persisted at the time of the appointment. Three potential genotypes of ACE2 *rs2285666* and *rs2074192*, TMPRSS2 *rs12329760* and *rs2070788*, and ACE1 *rs1799752* polymorphisms were identified from saliva samples. Further, methylation levels at five different locations (CpG) of dinucleotides in the ACE2 promoter were quantified using bisulfited pyrosequencing. Differences in the methylation percentage (%) of each CpG according to the genotype of the five polymorphisms were analyzed. Participants were evaluated up to 17.8 (SD: 5.2) months after hospital discharge. Eighty-eight percent (88.1%) of patients reported at least one post-COVID symptom (mean number of post-COVID symptoms: 3.0; SD: 1.9). Overall, we did not observe significant differences in the methylation levels of the ACE2 promoter according to the genotype of ACE2 *rs2285666* and *rs2074192*, TMPRSS2 *rs12329760* and *rs2070788*, or ACE1 *rs1799752* single nucleoid polymorphisms. This study did not find an association between genetics (genotypes of five COVID-19-associated polymorphisms) and epigenetics (methylation levels of the ACE2 promoter) in a cohort of COVID-19 survivors with post-COVID-19 condition who were hospitalized during the first wave of the pandemic.

## 1. Introduction

The severe acute respiratory syndrome coronavirus 2 (SARS-CoV-2) has provoked the biggest healthcare crisis of this century due to the propagation of the coronavirus disease, 2019 (COVID-19). By 2025, nearly 777 million individuals had been infected and more than 7 million are deceased because of SARS-CoV-2 infection [1].

In recent years, there has been an increase in knowledge about the role of genetics and epigenetics in COVID-19 severity. Genetics can be defined as the molecular processes able to regulate gene expression by determining its DNA sequence, whereas epigenetics can be defined as molecular processes able to regulate gene expression but without altering the DNA sequence [2]. Evidence has observed the involvement of surface receptors for S1 of the angiotensin-converting enzyme 2 (ACE2), angiotensin-converting enzyme 1 (ACE1), as well as transmembrane protease serine-2 (TMPRSS2) receptors as potential host for SARS-CoV-2 infection [3]. From a genetic viewpoint, several single studies have investigated the association of different single nucleotide polymorphisms (SNPs) of ACE1 (e.g., rs1799752), ACE2 (e.g., rs2285666), or TMPRSS2 (e.g., rs12329760) genes with the severity of COVID-19 [4,5,6]. The results showed that the presence of specific alleles (e.g., T allele of the ACE2 *rs2285666* or the T allele of the TMPRSS2 *rs12329760* polymorphisms) is associated with a lower severity of COVID-19 disease; however, when pooling current data into meta-analyses, the results are not conclusive due to their heterogeneity [4,5,6]. The presence of ACE2 and TMPRSS2 receptors in multiple organs can explain the plethora of symptoms that patients exhibit during the acute COVID-19 phase. From an epigenetic point of view, hypomethylation of the ACE2 promoter has been found to improve the expression of this receptor and, accordingly, a consequent increase in SARS-CoV-2 infection [7]. In fact, a pattern of hypomethylation in the ACE2 gene promoter has been identified in subjects who were infected by SARS-CoV-2, but this pattern was dependent of individual variables, including sex, age, body mass index, smoking, and the presence of comorbidities [8]. This variability could be expected since methylation patterns are highly variable with time and only represent a cross-sectional timepoint view of an individual.

Mortality is not the only problem associated with SARS-CoV-2 infection. A second healthcare problem associated with COVID-19 is the development of long-lasting symptoms once the acute infection has surpassed. The presence of long-lasting symptoms has been defined as long COVID [9] or post-COVID-19 condition [10]. Current evidence reveals that long COVID is a heterogeneous condition since more than 100 symptoms have been attributed to SARS-CoV-2 [11]. This plethora of symptoms can explain discrepancies in the prevalence rates of post-COVID-19 condition identified in the literature. For instance, Sk Abd Razak et al. reported a worldwide prevalence of post-COVID-19 condition of up to 41.8% of survivors [12]. On the other hand, the Global Burden of Disease Long COVID study reported a prevalence of post-COVID symptoms of 15% one year after the infection [13]. Thus, most of the published meta-analyses have reported that 25% of COVID-19 survivors could experience any type of post-COVID symptom one [14,15] and two [16,17] years after the infection. In addition, the prevalence of each post-COVID symptom is also different across epidemiological studies, supporting a multifactorial pathogenesis of long COVID. Thus, it could be hypothesized that this plethora of post-COVID symptoms could be related to the multisystemic location of ACE2 and TMPRSS2 receptors.

The heterogeneity of post-COVID-19 condition has led to different hypotheses explaining this plethora of symptoms. Among those potential factors hypothesized to be involved in the pathogenesis of post-COVID-19 condition, genetics and epigenetics have emerged as promising mechanisms; nevertheless, evidence on this topic is still in its infancy if compared with research of their role at the acute phase of infection [18]. Our research group identified that four polymorphisms associated with a more severe form of COVID-19 disease (ACE2 *rs2285666* and *rs2074192*, TMPRSS2 *rs12329760* and *rs2070788*) were not associated with the presence of specific post-COVID symptoms, including fatigue, dyspnea, or gastrointestinal problems [19]. The association of epigenetics and post-COVID-19 condition is heterogenous. Two studies including a small case series of patients with long COVID identified methylation changes at one year after the initial SARS-CoV-2 infection [20,21], whereas our research group identified that the methylation levels of the ACE2 promoter were not related to the presence of long COVID symptoms in a cohort of previously hospitalized COVID-19 survivors [22].

Genetic and epigenetic variability is commonly considered two independent mechanisms; however, it would be reasonable to expect that they can be intrinsically interconnected in their relationship with phenotypic variation [2]. No study has previously investigated whether an association between the methylation levels of the ACE2 promoter and these COVID-19-associated polymorphisms exists. We present a secondary analysis of our previous studies [19,22] showing data not published before. Therefore, the current study aimed to investigate if the DNA methylation levels of the ACE2 promoter are different depending on the genotype of five COVID-19-related polymorphisms: ACE2 *rs2285666* and *rs2074192*, TMPRSS2 *rs12329760* and *rs2070788*, and ACE1 *rs1799752*. We hypothesized that the methylation levels of the ACE2 promoter would be associated with specific genotypes of ACE2 polymorphisms, but not with TMPRSS2 or ACE1 genotypes.

## 2. Results

As previously described [19,22], from a sample of 330 individuals who had been hospitalized during the first wave of the pandemic due to COVID-19 that were invited to participate, a total of 279 (51.3% male, mean age: 56.0 ± 12.5 years old) fulfilled all the inclusion criteria and agreed to participate. The participants were assessed up to 17.8 (SD 5.2) months after hospitalization. At the time of this study, 246 (88.1%) survivors reported at least one post-COVID symptom (mean number of post-COVID symptoms/patient: 3.0; SD: 1.9). Table 1 summarizes the pre-COVID, COVID-19 onset, and post-COVID data of the total sample.

As previously described [19], the genotype distributions deviated from that expected based on the Hardy–Weinberg equilibrium (*p* < 0.01). Overall, we did not observe significant differences in the methylation levels of the ACE2 promoter according to the genotype of ACE2 *rs2285666* (Table 2) and *rs2074192* (Table 3), TMPRSS2 *rs12329760* (Table 4) and *rs2070788* (Table 5), or ACE1 *rs1799752* (Table 6) polymorphisms at any CpG location.

The inclusion of sex into the analysis did not reveal a significant sex * polymorphism interaction for methylation percentages of the ACE2 promoter at any CpG site: sex * ACE2 *rs2285666* (CpG1: F = 0.498, *p* = 0.701; CpG2: F = 0.104, *p* = 0.901; CpG3: F = 0.386, *p* = 0.680; CpG4: F = 0.739, *p* = 0.479; CpG5: F = 0.013, *p* = 0.987); sex * ACE2 *rs2074192* (CpG1: F = 0.123, *p* = 0.884; CpG2: F = 0.515, *p* = 0.601; CpG3: F = 0.853, *p* = 0.408; CpG4: F = 0.446, *p* = 0.641; CpG5: F = 0.468, *p* = 0.627); sex * TMPRSS2 *rs12329760* (CpG1: F = 0.399, *p* = 0.528; CpG2: F = 0.056, *p* = 0.814; CpG3: F = 0.059, *p* = 0.808; CpG4: F = 0.455, *p* = 0.501; CpG5: F = 0.054, *p* = 0.817); sex * TMPRSS2 *rs2070788* (CpG1: F = 1.119, *p* = 0.329; CpG2: F = 1.118, *p* = 0.330; CpG3: F = 1.235, *p* = 0.310; CpG4: F = 1.290, *p* = 0.278; CpG5: F = 0.791, *p* = 0.455); sex * ACE1 *rs1799752* (CpG1: F = 0.051, *p* = 0.822; CpG2: F = 1.195, *p* = 0.316; CpG3: F = 1.540, *p* = 0.305; CpG4: F = 1.556, *p* = 0.301; CpG5: F = 0.365, *p* = 0.546).

## 3. Discussion

The mechanisms behind the development of post-COVID-19 condition are complex, and different processes, including genetics and epigenetics, are proposed [18]. This study did not observe an association between the methylation levels of the ACE2 promoter and the genotypes of five COVID-19-associated polymorphisms (e.g., ACE2 *rs2285666* and *rs2074192*, TMPRSS2 *rs12329760* and *rs2070788*, ACE1 *rs1799752*) in a cohort of previously hospitalized COVID-19 survivors during the first wave of the pandemic with post-COVID symptoms. Evidence supports that the ACE2 and TMPRSS2 receptors are the path for the invasion of SARS-CoV-2 virus into the host cell and the aggressiveness of infection [23]. Previous single studies observed an association of these polymorphisms with a higher risk of SARS-CoV-2 infection or with a more severe form of COVID-19 disease [24,25,26,27,28]; however, the results from meta-analyses are not consistent due to the heterogeneity in the designs [4,5,6]. Data on a potential genetic influence on post-COVID symptomatology is scarce when compared with data at the acute COVID-19 phase [19].

Thus, the effects of SARS-CoV-2 on epigenetics have mainly focused on the analysis of DNA methylation changes during the acute phase of the infection [8]. In this direction, preliminary research has identified 35 candidate genes in DNE methylation analyses that can potentially serve as markers for SARS-CoV-2 infection [29]. Thus, differentially methylated sites were in the promoter regions of those genes for which functions are associated with biological processes such as IL-13 activation, T-helper differentiation, neuropeptide activity, and the release of chemokines, suggesting a connection between the progression of COVID-19 and inflammation [29]. Further, it has been found that females show the specific downregulation of the methylation of the ACE2 gene, suggesting a potential link between angiotensin II metabolism and hormonal changes influenced by chromosome dosage [30]. However, a recent review found that the DNA methylation changes in people with post-COVID-19 condition are heterogeneous [31]. Two small studies have reported the presence of a hypomethylation pattern in ten [20] and fifteen [21] patients with post-COVID-19 condition, whereas our research group did not identify an association between the methylation levels of the ACE2 promoter and specific post-COVID symptoms, e.g., fatigue, concentration loss, or fatigue [22]. In fact, the authors of the review in [31] concluded that while the included studies often lacked detailed patient characteristics and had small sample sizes, the epigenetic mechanisms in long COVID should be investigated. Further knowledge in epigenetics could help to identify specific mechanisms to facilitate patient subgrouping and to improve the way for personalized treatments [31].

An important topic to consider is that genetics are stable and not reversible, whereas epigenetics are fluctuating and reversible. In fact, no timeframe can currently be made for identifying DNA methylation changes. It could be hypothesized that SARS-CoV-2 could lead to DNA methylation changes in particular genes during the acute phase of infection, but these changes could reverse with time and, accordingly, would not be associated with the development of post-COVID symptoms. Another hypothesis would be that these epigenetic changes can be pre-determined by a genetic influence. It is at this point where this study investigated the association between genetics and epigenetics in individuals with post-COVID-19 condition. In fact, this is the first study investigating whether epigenetics (e.g., DNA methylation levels) could be determined by genetics (e.g., any genotype of SNP of the same promoter). The results did not reveal an association between the methylation levels of the ACE2 promoter and the genotype of COVID-19-associated polymorphisms (e.g., ACE2 *rs2285666* and *rs2074192*, TMPRSS2 *rs12329760* and *rs2070788*, ACE1 *rs1799752*) in a cohort of previously hospitalized COVID-19 survivors with post-COVID-19 condition. Nevertheless, it should be considered that we collected genetic and epigenetic factors on saliva samples. Since DNA methylation is a tissue-specific pattern that can be reversible, it is possible that the use of salivary samples instead of blood could lead to a potential variability in the results. Studies using blood samples are now needed to further confirm or refute the current results.

Although this is the first study integrating epigenetics and genetics in patients with post-COVID symptoms, the results should be considered according to some limitations. First, our sample included individuals who had been previously hospitalized by an acute SARS-CoV-2 infection during the first wave of the pandemic, so they were infected with a historical strain; therefore, the current results cannot be extrapolated to individuals infected with other variants of concern. Second, all the sample was recruited from a single geographic location (Madrid, Spain). Third, the participants were hospitalized due to COVID-19 and, accordingly, the current results should not be applied to non-hospitalized patients. Thus, all participants were infected and developed post-COVID symptoms before receiving any COVID-19 vaccine dose. Since vaccination can decrease the risk of long COVID if administered before SARS-CoV-2 infection [32], we do not know if methylation changes would be affected by vaccination status. Fourth, the cross-sectional nature of our study limits the extrapolation of the changes to longitudinal scenarios since methylation changes are fluctuating, whereas genetics are stable. In fact, we did not collect pre-COVID genetic and epigenetic data; therefore, we do not know if the observed findings are directly related to SARS-CoV-2 infection. Thus, it should be considered that we collect data up to 18 months after hospitalization; accordingly, we cannot exclude the influence of surrounding environmental factors, e.g., nutrition, physical activity, or exposure to other toxins, that can affect methylation levels. Finally, it should be recognized that we focused on specific genes, particularly those related to SARS-CoV-2 trophism. Population-based studies including more genes could open up a new research line to be associated with methylation levels.

## 4. Materials and Methods

### 4.1. Participants

A secondary analysis including a cohort of COVID-19 survivors hospitalized at four urban hospitals in Madrid (Spain) during the first wave of the pandemic (from March to May 2020) was conducted [19,22]. As previously described, all participants received a diagnosis of SARS-CoV-2 infection by a reverse transcription–polymerase chain reaction (RT-PCR) assay of the nasopharyngeal and an oral swab sample, as well as clinical/radiological findings at their hospital admission [19,22]. This study was approved by the Institutional Ethics Committees of all institutions recruiting participants (HUFA 20/126; HCSC20/495E, HSO25112020; HUIL/092-20; URJC0907202015920). All participants signed a written informed consent form prior to us collecting any of their data.

As previously described [19,22], the current study included retrospective and prospective data collection. Medical records were used to collect demographic, clinical, and hospitalization data. The presence of post-COVID symptoms was obtained from individual face-to-face appointments conducted by a healthcare professional [19,22]. Data on post-COVID symptoms have already been published and will not be presented here again [19,22].

### 4.2. Biological Sample Collection

Unstimulated whole saliva samples were collected from each participant into collection tubes according to standardized procedures at the face-to-face appointments [19,22]. As previously described [19,22], patients were seated and relaxed during saliva collection. Thus, all samples were collected during the morning. Participants avoided eating, drinking, or chewing gum for at least 1 h before the saliva collection. Saliva samples were centrifuged at 3000 rpm for 15 min to obtain the cell sediment self-collection procedure immediately after the collection and stored at −20 °C until the analysis [19,20]. Saliva samples were collected instead of whole blood because it is a non-invasive and stress-free assessment method and because salivary DNA is equivalent in quantity and purity to blood DNA [33]. Saliva samples are commonly used more frequently to assess DNA methylation in the former literature [34].

### 4.3. Genome DNA Extraction

The genomic DNA extraction procedure has been previously described [19,22]. Briefly, genomic DNA was extracted from 500 μL of saliva using a MagMAX™ DNA Multi-Sample Ultra 2.0 Kit (Thermo Fisher Scientific Inc., Hemel Hempstead, Hertfordshire, UK) according to the manufacturer’s protocol. We automatically extracted DNA using the King Fisher Flex purification robot (Thermo Fisher). The resulting DNA was assessed for purity and concentration using Quant-iT™ PicoGreen™ dsDNA reagent” (Thermo Fisher). DNA was diluted to 5 ng/μL using 1×Tris-EDTA (TE) buffer (Sigma-Aldrich, Dorset, UK). The qPCR reaction mixtures of 10 μL contained a total of 10 ng gDNA as a PCR template, 1× TaqMan Gene Expression PCR Master Mix, and 0.6× Genotyping TaqMan-probe assay [19,22].

### 4.4. Single Nucleotide Polymorphism Genotyping

Single nucleotide polymorphism (SNP) genotyping was conducted with taqMan^®^ Predesigned SNP Genotyping Assays (Thermo Fisher Scientific Inc., Hertfordshire, UK). Real-time PCR plates were run in the Quantstudio 12K Flex System (Thermo Fisher) of the Genomics Unit (Madrid Science Park Foundation, Spain) under standard conditions (95° for 10 min and 40 two-step cycles consisting of 95 °C for 15 s and 60 °C for 1 min) and analyzed with the Genotyping App of Thermo Fisher Cloud. The identification of each genotype was conducted by using specific fluorescent dyes.

The possible alleles of the ACE2 *rs2285666* SNP led to the following genotypes (C/C, C/T, T/T) derived from a C → T substitution at the following sequence:

TAATCACTACTAAAAATTAGTAGC [C/T] TACCTGGTTCAAGTAATAAGCATTC

The possible alleles of the ACE2 *rs2074192* led to the following genotypes (C/C, C/T, T/T) derived from a C → T substitution at the following sequence:

GTGGAAATGTATAAATGGTTGG [C/T] ATTTATTCATTTGTGACTGCTG

The possible alleles of the TMPRSS2 *rs12329760* led to the following genotypes (C/C, C/T, T/T) derived from a C → T substitution at the following sequence:

CTTCCTCTGAGATGAGTACA [C/T] CTGAAGGATGAAGTTTGGTC

The possible alleles of the TMPRSS2 *rs2070788* led to the following genotypes (G/G, G/A, A/A) derived from a G → A substitution at the following sequence:

TGTTGTCTGTATGGCCTAGAC [G/A] CTTTTGAGAAGGATATAA

The possible alleles of the ACE1 *rs1799752* (the minor allele -I allele- and the common allele -D allele-) led to the following genotypes (D/D, D/I, I/I) derived from the sequence:

CCCATTTCTCTAGACCTGCTGCCT [-/ALU] ATACAGTCACTTTTATGTGGTTTC

### 4.5. Methylation Profiling

As previously described [22], bisulfite conversion, amplification of target sequences, and sequencing were performed at Fundación Parque Científico de Madrid (c/Faraday 7, Madrid, Spain).

Genomic DNA was bisulfite-converted using the Epitech Fast 96 Bisulfite Kit (Cat n° 50959720, Werfen, Barcelona, Spain) following the manufacturer’s instructions [20]. Analyses of ACE2 promoter methylation were amplified using tailed oligos, i.e., a unique amplicon-specific part, fused to 5′-tail comprising sequences necessary for sequencing reactions. A web-based program (http://www.urogene.org/methprimer, accessed on 1 December 24) was used to identify non-cytosine-phosphate-guanine (CpG) sites in the ACE2 promoter. Accordingly, five CpG sites of interest (CpG1, CpG2, CpG3, CpG4, CpG5) within the ACE2 promoter were selected as previously described [35,36].

Following ACE2-specific amplification, amplification products were purified from agarose gels, titrated, and diluted for further processing. NGS libraries were made using a collection of Illumina-compatible PCR primers, including a 10 bp barcode identifier (MID) used to identify each sample within the pool [20]. Finally, samples were subjected to Illumina sequencing in MiSeq (2 × 250 reads). The sequencing run yielded over 840,000 filtered, quality reads, an average of about 1800 reads per amplicon per sample (range 500 to 5000). The percentage of methylation per sample within each CpG was calculated as the percentage C/C+T and used in the analysis; the mean value of all CpG dinucleotides per amplicon was calculated to represent the methylation value of a particular locus. We analyzed the methylation percentage (%) of each position (CpG1, CpG2, CpG3, CpG4, CpG5) separately.

Sequencing depth and biological dispersion were carefully considered and controlled to ensure the robustness and reliability of the results obtained. For that purpose, we aimed for a minimum sequencing depth of 100× to balance between cost and data quality, ensuring that each genomic region was sequenced multiple times to reduce the likelihood of false positives and to increase the reliability of our results. Thus, biological dispersion was accounted for by including several biological replicates. This approach allowed us to capture the natural variability in the samples and provided a robust statistical framework for identifying true genetic variations. Additionally, we employed rigorous quality control measures to filter out low-quality reads and potential sequencing artifacts, further enhancing the accuracy of our genotyping analysis.

### 4.6. Statistical Analysis

Data were collected with STATA 16.1 and processed using Python’s library pandas 0.25.3. The Shapiro–Wilk test was used to assess the assumption of normality. Since all data did not follow normal distribution, medians (interquartile ranges) are presented for quantitative data and number of cases (percentages) is presented for categorical data. Thus, non-parametric tests were used. Differences in the methylation percentage (%) at each CpG according to genotype were analyzed with non-parametric Kruskal–Wallis tests. In addition, a one-way analysis of variance (ANOVA) with sex and polymorphism genotype was conducted to determine the effect of sex on methylation percentages at each CpG site. The level of significance was set at 0.05 a priori, with the *p*-values from all tests being corrected (Holm–Bonferroni correction).

## 5. Conclusions

This study did not find an association between the methylation levels of the ACE2 promoter and the genotypes of five COVID-19-associated polymorphisms (e.g., ACE2 *rs2285666* and *rs2074192*, TMPRSS2 *rs12329760* and *rs2070788*, ACE1 *rs1799752*) in individuals who had been previously hospitalized due to COVID-19 during the first wave of the pandemic and experiencing post-COVID symptoms.

## Figures and Tables

**Table 1:** Pre-infection data, COVID-19-associated onset symptoms, and post-COVID symptoms of the total sample.

	Cohort (n = 279)
Age, mean (SD), years	56.0 (12.5)
Gender, female n (%)	136 (48.7%)
Weight, mean (SD), kg.	81.0 (16.5)
Height, mean (SD), cm.	167.0 (9.5)
Medical co-morbidities, n (%)	
Hypertension	95 (34.0%)
Obesity	85 (30.4%)
Asthma	31 (11.0%)
Diabetes	29 (10.0%)
Cardiovascular diseases	20 (7.0%)
Chronic obstructive pulmonary disease	5 (1.8%)
COVID-19 onset symptoms, n (%)	
Fever	202 (72.4%)
Dyspnea	102 (36.6%)
Myalgia	140 (49.9%)
Cough	96 (34.4%)
Headache	87 (31.1%)
Diarrhea	54 (19.3%)
Anosmia/hyposmia	63 (22.6%)
Ageusia/hypogeusia	65 (23.2%)
Throat pain	32 (11.5%)
Number post-COVID symptoms, mean (SD)	3.0 (2.0)
Post-COVID symptoms, n (%)	
Fatigue	174 (62.3%)
Pain symptoms	112 (40.1%)
Memory loss	87 (31.1%)
Hair loss	70 (25.1%)
Concentration loss	42 (15.0%)
Cognitive blunting—brain fog	41 (14.7%)
Ocular disorders	41 (14.7%)
Dyspnea	36 (13.0%)
Skin rashes	36 (13.0%)
Anosmia	29 (10.4%)
Gastrointestinal disorders	25 (9.0%)
Ageusia	21 (7.5%)
Days in hospital, mean (SD)	8.0 (7.7)

**Table 2:** Number of post-COVID symptoms and methylation percentages expressed as median (interquartile range) at each CpG site according to the ACE2 *rs2285666* genotype (n = 279).

	C/C (n = 189)	C/T (n = 43)	T/T (n = 47)	*p*-Value
Number post-COVID symptoms	3.0 (3.0)	4.0 (2.0)	3.0 (3.0)	0.404
CpG1 methylation (%)	94.3 (2.6)	94.5 (1.9)	94.5 (1.5)	0.888
CpG2 methylation (%)	40.3 (12.2)	39.4 (13.1)	42.4 (8.9)	0.759
CpG3 methylation (%)	44.1 (13.5)	45.4 (13.7)	43.9 (11.8)	0.499
CpG4 methylation (%)	46.1 (9.7)	47.5 (9.3)	46.2 (9.6)	0.325
CpG5 methylation (%)	0.6 (0.3)	0.55 (0.4)	0.6 (0.3)	0.604

**Table 3:** Number of post-COVID symptoms and methylation percentages expressed as median (interquartile range) at each CpG site according to the ACE2 *rs2074192* genotype (n = 279).

	C/C (n = 128)	C/T (n = 63)	T/T (n = 88)	*p*-Value
Number post-COVID symptoms	3.0 (2.5)	3.0 (3.0)	3.0 (3.0)	0.933
CpG1 methylation (%)	94.3 (2.8)	94.3 (2.5)	94.3 (2.1)	0.530
CpG2 methylation (%)	40.5 (12.0)	40.9 (9.8)	39.8 (11.9)	0.538
CpG3 methylation (%)	43.6 (13.8)	44.6 (12.0)	43.8 (12.0)	0.367
CpG4 methylation (%)	46.1 (9.6)	47.5 (8.8)	46.5 (11.1)	0.435
CpG5 methylation (%)	0.6 (0.3)	0.55 (0.4)	0.6 (0.4)	0.405

**Table 4:** Number of post-COVID symptoms and methylation percentages expressed as median (interquartile range) at each CpG site according to the TMPRSS2 *rs12329760* genotype (n = 279).

	C/C (n = 213)	C/T (n = 62)	T/T (n = 4)	*p*-Value
Number post-COVID symptoms	3.0 (3.0)	3.0 (2.9)	3.0 (3.7)	0.955
CpG1 methylation (%)	94.4 (2.5)	94.0 (2.1)	95.2 (2.4)	0.570
CpG2 methylation (%)	40.6 (11.2)	40.0 (11.4)	40.0 (12.8)	0.852
CpG3 methylation (%)	43.8 (13.0)	44.2 (12.5)	44.6 (10.5)	0.929
CpG4 methylation (%)	46.5 (10.1)	45.6 (8.5)	45.4 (9.3)	0.851
CpG5 methylation (%)	0.55 (0.35)	0.6 (0.25)	0.65 (0.4)	0.517

**Table 5:** Number of post-COVID symptoms and methylation percentages expressed as median (interquartile range) at each CpG site according to the TMPRSS2 *rs2070788* genotype (n = 279).

	A/A (n = 77)	A/G (n = 140)	G/G (n = 62)	*p*-Value
Number post-COVID symptoms	3.0 (3.5)	3.7 (3.0)	3.0 (2.8)	0.592
CpG1 methylation (%)	94.4 (2.1)	94.0 (2.5)	94.6 (1.7)	0.857
CpG2 methylation (%)	39.8 (11.2)	39.7 (12.7)	42.7 (9.3)	0.205
CpG3 methylation (%)	43.8 (13.3)	43.1 (13.5)	46.0 (11.4)	0.308
CpG4 methylation (%)	45.8 (9.0)	46.5 (9.9)	46.5 (9.9)	0.796
CpG5 methylation (%)	0.6 (0.35)	0.6 (0.3)	0.55 (0.3)	0.551

**Table 6:** Number of post-COVID symptoms and methylation percentages expressed as median (interquartile range) at each CpG site according to the ACE1 *rs1799752* genotype (n = 279).

	D/D (n = 104)	D/I (n = 170)	I/I (n = 5)	*p*-Value
Number post-COVID symptoms	3.0 (3.5)	3.0 (2.0)	3.5 (4.0)	0.296
CpG1 methylation (%)	94.4 (2.1)	94.3 (2.55)	94.0 (1.5)	0.920
CpG2 methylation (%)	41.5 (11.1)	39.4 (12.0)	38.9 (11.7)	0.360
CpG3 methylation (%)	45.4 (11.9)	43.4 (10.5)	40.5 (10.8)	0.307
CpG4 methylation (%)	46.7 (9.0)	46.5 (9.9)	45.5 (9.3)	0.434
CpG5 methylation (%)	0.6 (0.4)	0.55 (0.3)	0.65 (0.2)	0.466

## Data Availability

All data derived from this study are presented in the text.

## References

[1] World Health Organization (2024). COVID-19 Case Dashboard. https://data.who.int/dashboards/covid19/cases.

[2] Capp J.P. (2021). Interplay between genetic, epigenetic, and gene expression variability: Considering complexity in evolvability. Evol. Appl..

[3] Adli A., Rahimi M., Khodaie R., Hashemzaei N., Hosseini S.M. (2022). Role of genetic variants and host polymorphisms on COVID-19: From viral entrance mechanisms to immunological reactions. J. Med. Virol..

[4] Gupta K., Kaur G., Pathak T., Banerjee I. (2022). Systematic review and meta-analysis of human genetic variants contributing to COVID-19 susceptibility and severity. Gene.

[5] Pecoraro V., Cuccorese M., Trenti T. (2023). Genetic polymorphisms of ACE1, ACE2, IFTM3, TMPRSS2 and TNFα genes associated with susceptibility and severity of SARS-CoV-2 infection: A systematic review and meta-analysis. Clin. Exp. Med..

[6] Saengsiwaritt W., Jittikoon J., Chaikledkaew U., Udomsinprasert W. (2022). Genetic polymorphisms of ACE1, ACE2, and TMPRSS2 associated with COVID-19 severity: A systematic review with meta-analysis. Rev. Med. Virol..

[7] Faramarzi A., Safaralizadeh R., Dastmalchi N., Teimourian S. (2022). Epigenetic-related effects of COVID-19 on human cells. Infect. Disord. Drug Targets.

[8] Daniel G., Paola A.R., Nancy G., Fernando S.O., Beatriz A., Zulema R., Julieth A., Claudia C., Adriana R. (2022). Epigenetic mechanisms and host factors impact ACE2 gene expression: Implications in COVID-19 susceptibility. Infect. Genet. Evol..

[9] Fernández-de-las-Peñas C. (2021). Long COVID: Current definition. Infection.

[10] Soriano J.B., Murthy S., Marshall J.C., Relan P., Diaz J.V., WHO Clinical Case Definition Working Group on Post-COVID-19 Condition (2022). A clinical case definition of post-COVID-19 condition by a Delphi consensus. Lancet Infect. Dis..

[11] Hayes L.D., Ingram J., Sculthorpe N.F. (2021). More Than 100 Persistent Symptoms of SARS-CoV-2 (Long COVID): A scoping review. Front. Med..

[12] Sk Abd Razak R., Ismail A., Abdul Aziz A.F., Suddin L.S., Azzeri A., Sha’ari N.I. (2024). Post-COVID syndrome prevalence: A systematic review and meta-analysis. BMC Public Health.

[13] Wulf Hanson S., Abbafati C., Aerts J.G., Al-Aly Z., Ashbaugh C., Ballouz T., Blyuss O., Bobkova P., Bonsel G., Global Burden of Disease Long COVID Collaborators (2022). Estimated global proportions of individuals with persistent fatigue, cognitive, and respiratory symptom clusters following symptomatic COVID-19 in 2020 and 2021. JAMA.

[14] Chen C., Haupert S.R., Zimmermann L., Shi X., Fritsche L.G., Mukherjee B. (2022). Global prevalence of post COVID-19 condition or long COVID: A meta-analysis and systematic review. J. Infect. Dis..

[15] Han Q., Zheng B., Daines L., Sheikh A. (2022). Long-term sequelae of COVID-19: A systematic review and meta-analysis of one-year follow-up studies on post-COVID symptoms. Pathogens.

[16] Fernández-de-las-Peñas C., Notarte K.I., Macasaet R., Velasco J.V., Catahay J.A., Therese Ver A., Chung W., Valera-Calero J.A., Navarro-Santana M. (2024). Persistence of post-COVID symptoms in the general population two years after SARS-CoV-2 infection: A systematic review and meta-analysis. J. Infect..

[17] Rahmati M., Udeh R., Yon D.K., Lee S.W., Dolja-Gore X., McEVoy M., Kenna T., Jacob L., López Sánchez G.F., Koyanagi A. (2023). A systematic review and meta-analysis of long-term sequelae of COVID-19 2-year after SARS-CoV-2 infection: A call to action for neurological, physical, and psychological sciences. J. Med. Virol..

[18] Fernández-de-las-Peñas C., Raveendran A.V., Giordano R., Arendt-Nielsen L. (2023). Long COVID or post-COVID-19 condition: Past, present and future research directions. Microorganisms.

[19] Fernández-de-las-Peñas C., Arendt-Nielsen L., Díaz-Gil G., Gómez-Esquer F., Gil-Crujera A., Gómez-Sánchez S.M., Ambite-Quesada S., Palomar-Gallego M.A., Pellicer-Valero O.J., Giordano R. (2022). Genetic association between ACE2 (rs2285666 and rs2074192) and TMPRSS2 (rs12329760 and rs2070788) polymorphisms with post-COVID symptoms in previously hospitalized COVID-19 survivors. Genes.

[20] Nikesjö F., Sayyab S., Karlsson L., Apostolou E., Rosén A., Hedman K., Lerm M. (2022). Defining post-acute COVID-19 syndrome (PACS) by an epigenetic biosignature in peripheral blood mononuclear cells. Clin. Epigenetics.

[21] Balnis J., Madrid A., Hogan K.J., Drake L.A., Adhikari A., Vancavage R., Singer H.A., Alisch R.S., Jaitovich A. (2023). Whole-Genome methylation sequencing reveals that COVID-19-induced epigenetic dysregulation remains 1 year after hospital discharge. Am. J. Respir. Cell Mol. Biol..

[22] Fernández-de-las-Peñas C., Díaz-Gil G., Gil-Crujera A., Gómez-Sánchez S.M., Ambite-Quesada S., Torres-Macho J., Ryan-Murua P., Franco-Moreno A., Pellicer-Valero O.J., Arendt-Nielsen L. (2024). DNA Methylation levels of the ACE2 Promoter are not associated with post-COVID-19 symptoms in individuals who had been hospitalized due to COVID-19. Microorganisms.

[23] Zipeto D., Palmeira J.D.F., Argañaraz G.A., Argañaraz E.R. (2020). ACE2/ADAM17/TMPRSS2 interplay may be the main risk factor for COVID-19. Front. Immunol..

[24] Srivastava A., Bandopadhyay A., Das D., Pandey R.K., Singh V., Khanam N., Srivastava N., Singh P.P., Dubey P.K., Pathak A. (2020). Genetic association of ACE2 rs2285666 polymorphism with COVID-19 spatial distribution in India. Front. Genet..

[25] Ravikanth V., Sasikala M., Naveen V., Latha S.S., Parsa K.V.L., Vijayasarathy K., Amanchy R., Avanthi S., Govardhan B., Rakesh K. (2021). A variant in TMPRSS2 is associated with decreased disease severity in COVID-19. Meta Gene.

[26] Hou Y., Zhao J., Martin W., Kallianpur A., Chung M.K., Jehi L., Sharifi N., Erzurum S., Eng C., Cheng F. (2020). New insights into genetic susceptibility of COVID-19: An ACE2 and TMPRSS2 polymorphism analysis. BMC Med..

[27] Martínez-Gómez L.E., Herrera-López B., Martinez-Armenta C., Ortega-Peña S., Camacho-Rea M.D.C., Suarez-Ahedo C., Vázquez-Cárdenas P., Vargas-Alarcón G., Rojas-Velasco G., Fragoso J.M. (2022). ACE and ACE2 gene variants are associated with severe outcomes of COVID-19 in men. Front. Immunol..

[28] Wulandari L., Hamidah B., Pakpahan C., Damayanti N.S., Kurniati N.D., Adiatmaja C.O., Wigianita M.R., Soedarsono, Husada D., Tinduh D. (2021). Initial study on TMPRSS2 p. Val160Met genetic variant in COVID-19 patients. Hum. Genom..

[29] Zhou S., Zhang J., Xu J., Zhang F., Li P., He Y., Wu J., Wang C., Wang X., Zhang W. (2021). An epigenome-wide DNA methylation study of patients with COVID-19. Ann. Hum. Genet..

[30] Joubert B.R., Felix J.F., Yousefi P., Bakulski K.M., Just A.C., Breton C., Reese S.E., Markunas C.A., Richmond R.C., Xu C.J. (2016). DNA methylation in newborns and maternal smoking in pregnancy: Genome-wide consortium meta-analysis. Am. J. Hum. Genet..

[31] Shekhar Patil M., Richter E., Fanning L., Hendrix J., Wyns A., Barrero Santiago L., Nijs J., Godderis L., Polli A. (2024). Epigenetic changes in patients with post-acute COVID-19 symptoms (PACS) and long-COVID: A systematic review. Expert Rev. Mol. Med..

[32] Watanabe A., Iwagami M., Yasuhara J., Takagi H., Kuno T. (2023). Protective effect of COVID-19 vaccination against long COVID syndrome: A systematic review and meta-analysis. Vaccine.

[33] Khare P., Raj V., Chandra S., Agarwal S. (2014). Quantitative and qualitative assessment of DNA extracted from saliva for its use in forensic identification. J. Forensic Dent. Sci..

[34] Nishitani S., Parets S.E., Haas B.W., Smith A.K. (2018). DNA methylation analysis from saliva samples for epidemiological studies. Epigenetics.

[35] Mikeska T., Felsberg J., Hewitt C.A., Dobrovic A. (2011). Analysing DNA methylation using bisulphite pyrosequencing. Methods Mol. Biol..

[36] Fan R., Mao S.Q., Gu T.L., Zhong F.D., Gong M.L., Hao L.M., Yin F.Y., Dong C.Z., Zhang L.N. (2017). Preliminary analysis of the association between methylation of the ACE2 promoter and essential hypertension. Mol. Med. Rep..

